# Nitroglycerin and Iloprost Improve Mitochondrial Function in Colon Homogenate Without Altering the Barrier Integrity of Caco-2 Monolayers

**DOI:** 10.3389/fmed.2018.00291

**Published:** 2018-10-16

**Authors:** Anna Herminghaus, Rebecca Eberhardt, Richard Truse, Jan Schulz, Inge Bauer, Olaf Picker, Christian Vollmer

**Affiliations:** Department of Anaesthesiology, University of Düsseldorf, Düsseldorf, Germany

**Keywords:** nitroglycerin, iloprost, mitochondrial respiration, barrier function, Caco-2 cells

## Abstract

Locally applied nitroglycerin [nitric oxide (NO) donor] and iloprost (analog of prostacyclin PGI_2_) improve regional gastric oxygenation and nitroglycerin preserves gastric mucosal barrier integrity. This suggests direct effects of these substances on oxygenation and barrier function. The aim of this study was to analyze the effect of iloprost and nitroglycerin on intestinal mitochondrial function and on mucosal barrier function *in vitro*. Mitochondrial oxygen consumption (respirometry) was determined in colon homogenates from 16 healthy rats before (baseline) and 15 min after incubation with nitroglycerin (25 and 250 μg/ml) and iloprost (0.1 and 1 μg/ml). State 2 (substrate-dependent oxygen consumption) and state 3 respiration (ADP-dependent oxygen consumption) were assessed and ADP/O ratio (ADP added/oxygen consumed) for complex I and II were calculated. For permeability measurement we used the Caco-2 monolayer. Fluorescein sulfonic acid (FS) (200 μg/ml) and the drugs were administered into the apical compartment of the transwell chamber. After 48 h, FS translocation was assessed as basolateral/apical FS. Both concentrations of nitroglycerin and iloprost reduced state 3 by stimulation via both complexes. Iloprost increased ADP/O ratio after stimulation via both complexes at both concentrations. Nitroglycerin increased ADP/O ratio at the higher concentration (250 μg/ml) after stimulation via complex I and at the lower concentration (25 μg/ml) via complex II. Neither nitroglycerin nor iloprost influenced FS translocation. Iloprost and nitroglycerin reduce the maximal mitochondrial respiration and improve the efficacy of oxidative phosphorylation in colon homogenates. Both drugs have no direct influence on mucosal barrier integrity of Caco-2 monolayers.

## Introduction

Microcirculatory alterations and mitochondrial dysfunction are considered to be the main pathophysiological mechanisms in organ dysfunction during critical illness like septic shock (1). Additionally, reduced oxygenation may lead to a disruption of gastric and intestinal barrier function (2, 3), thereby further inducing translocation of bacteria and bacterial toxins into portal. venous and local lymphatic circulation This second hit leads to a secondary, more refractory shock and additional multiorgan dysfunction (4). Thus, growing effort is made to develop strategies to improve splanchnic mucosal oxygenation, intestinal barrier integrity, and mitochondrial function. We could show that under hemorrhagic conditions, locally applied nitroglycerin and iloprost improve regional gastric oxygenation (1). Moreover, nitroglycerin attenuated the shock-induced impairment of the gastric mucosal barrier integrity. This suggests potential direct effects of both substances on barrier function and on oxygenation, possibly via modulation of mitochondrial function. The effect of both drugs on mitochondrial function in intestinal cells, however, remains unclear since studies on intestinal mitochondria are very rare, possibly because of the sophisticated tissue preparation.

Although the effect of nitric oxide (NO) and its derivatives such as nitroglycerin has been studied in other tissues like skeletal muscle, hepatocytes and brain nerve terminals, and astrocytes (5, 6) there is no data so far analyzing the effect on intestinal mitochondria. This is of particular interest since mitochondrial function for a given stimulus differs organ dependently (7, 8). Concerning iloprost, there is little evidence about effects on mitochondrial function. Iloprost exerts protective effects on hepatic mitochondria as reflected in increased ADP/O ratio after ischemic injury (9), but there is no data concerning the influence of iloprost on gastrointestinal mitochondrial function. Thus, the impact of nitroglycerin and iloprost on intestinal mitochondria remains unknown. Studies on intestinal mitochondria are necessary as it is an important organ to maintain barrier function and prevent translocation of bacteria and toxins into the blood and local lymph system thus preventing sepsis.

The local application of drugs is advantageous since it allows high drug concentrations in target cells without or with reduced negative side effects. Therefore, modulation of mitochondrial function in intestinal cells with nitroglycerin or iloprost could be a promising therapeutic option for many pathological states, e.g., associated with sepsis.

Modulation of mitochondrial function might affect tissue function, e.g., barrier function of intestinal cells, as well. It is well known that gastrointestinal epithelial permeability can be modulated by many different factors and drugs like tissue pH (10), adenosine 3′,5′-cyclic monophosphate (11), insulin (12), insulin-like growth factors (13), and cytokines (14).

It is conceivable that NO could also modulate enterocyte tight junctions. Results from recent studies suggest that NO suppresses degranulation of the mast cells and as a consequence reduces release of potential harmful mediators like histamine and platelet-activating factor, thereby protecting the barrier function (15). However, other investigators have shown that iNOS as a source for NO can disrupt tight junctions and hereby damage barrier function (16). Data on the direct effect of nitroglycerin and iloprost on intestinal barrier function- independent of changes in microcirculation- is lacking completely.

Therefore, the aim of this study was to analyze

1st the effect of iloprost and nitroglycerin on intestinal mitochondrial function and

2nd the direct impact of iloprost and nitroglycerin on mucosal barrier function.

## Materials and methods

### Animals

The study was approved from the Animal Ethics Committee of the University of Duesseldorf, Germany, and conducted in accordance with the Guide for the Care and Use of Laboratory Animals of the National Institutes of Health.

Male Wistar rats were purchased from the breeding facilities of the University of Düsseldorf (Germany) or from Janvier (France). They were kept at an artificial 12 h light/dark cycle at constant room temperature and humidity with free access to standard chow and tap water.

Rats were sacrificed by decapitation under deep sedation with sodium pentobarbital (90 mg/kg) and the organs were harvested.

### Preparation of colon homogenates

Freshly harvested colon from 16 male Wistar rats (270–350 g) was placed in 4°C cold isolation puffer, quickly opened and dried softly to remove remains of feces and mucus. After incubation with trypsin for 5 min on ice, tissue was placed in 4°C cold isolation buffer (200 mM mannitol, 50 mM sucrose, 5 mM KH_2_PO_4_, 5 mM morpholinepropanesulfonic acid (MOPS), 1 mM EDTA, pH 7.15) containing 20 mg/ml bovine serum albumin (BSA) and protease inhibitors (Complete®, Roche, Germany), minced into 2–3mm^3^ pieces and homogenized (Potter-Elvehjem, 5 strokes, 2000 rpm).

Protein concentration in the tissue homogenates was determined by the Lowry method (17) with bovine serum albumin as external standard.

### Measurement of mitochondrial respiratory rate

Mitochondrial oxygen consumption was measured at 30°C using a Clark-type electrode (model 782, Strathkelvin instruments, Glasgow, Scotland). Tissue homogenates were suspended in respiration medium (KCl 130 mM, K_2_HPO_4_ 5 mM, MOPS 20 mM, EGTA 2.5 mM, Na_4_P_2_O_7_ 1 μM, BSA 2%, pH 7.15) to yield a protein concentration of 6 mg/ml.

Mitochondrial state 2 respiration was recorded in the presence of either complex I substrates glutamate and malate (2.5 mM, G-M) or complex II substrate succinate (5 mM, S). Mitochondrial enzyme glutamate dehydrogenase oxidizes glutamate to α-ketoglutarate, a reaction leading to reduction of NAD+ to NADH, which serves as substrate for complex I. Oxidation of succinate is coupled with the reduction of FAD to FADH_2_ which serves as substrate for complex II.

The maximal mitochondrial respiration in state 3 was measured after addition of ADP (125 μM). ADP-dependent oxygen consumption was measured during state 3 (deltaO). ADP/O ratio calculated from the amount of ADP added and O_2_-consumption reflects the effectiveness of oxidative phosphorylation.

The solubility of oxygen was assumed to be 223 μmol ${\text{O}}_{2}^{*}$l^−1^ at 30°C. Respiration rates were expressed as nmol/min/mg protein, but presented as percentage of the baseline.

Mitochondria were checked for leakage by addition of cytochrome c (2.5 μM) and oligomycin (0.05 μg/ml). There was no increase in flux after addition of cytochrome c, indicating integrity of the outer mitochondrial membrane. When ATP synthesis was inhibited by oligomycin, the nonphosphorylating state was analogous to state 2, which is limited only by substrate concentration and reflects intrinsic uncoupling as compensation for the proton leak. These results indicate that the inner membrane was intact and mitochondria were not damaged through the preparation procedure.

### Effect of iloprost and nitroglycerin on mitochondrial function

Tissue homogenates were incubated for 15 min with iloprost (0.1 and 1 μg/ml) or nitroglycerin (25 and 250 μg/ml) at room temperature (kept at 21°C). Measurement of mitochondrial respiratory rates was performed immediately after the incubation period.

Baseline measurement was performed before addition of the respective substance. Data are presented as percentage of the control value (*n* = 8).

### Effects of iloprost or nitroglycerin on mucosal barrier function

Barrier function is influenced *in vivo* by a variety of confounders, e. g., microcirculation. To avoid potential microcirculatory effects of nitroglycerin or iloprost, we performed this study *in vitro*. We used Caco-2 cells on porous membranes as a model system of intact intestinal epithelium. These cells exert functional and anatomic similarities to absorptive intestinal enterocytes. Hence, this cell line is well established to study intestinal permeability *in vitro* (18, 19).

### Reagents

Minimal essential medium eagle (MEME) was purchased from Sigma-Aldrich (Munich, Germany), Dulbecco's Modified Eagle Medium (DMEM) from PAN Biotech (Aidenbach, Germany). Other cell culture reagents were obtained from Biochrom (Berlin, Germany) or Life Technologies (Darmstadt, Germany).

### Cell culture

The human colon adenocarcinoma cell line Caco-2 (European Collection of Cell Cultures No. 86010202) was kindly provided by Dr. H. Steinbrenner (Institute for Biochemistry and Molecular Biology I; Duesseldorf, Germany). Cells were cultivated as described before (20) in MEME [containing 10% fetal calf serum (FCS), 1% penicillin/streptomycin, 1% non-essential amino acids (MEM) and 1% L- alanyl-L glutamine-dipeptide (Glutamax^TM^)].

### Porous membrane supports

Porous membrane supports (Transwell-Clear, 12 mm diameter, 0.4 μm pore size, Corning Incorporated, NY, USA) were coated with 10 μg/cm^2^ collagen (Sigma-Aldrich, Munich, Germany). Caco-2 cells were seeded onto collagen-coated porous membrane supports at a density of 2 × 10^5^ cells/cm^2^ and placed in a Transwell chamber. Medium was changed twice a week. Cells were grown for at least 3 weeks to build a confluent monolayer. Experiments were performed between day 21 and 28 after seeding.

### Permeability measurement

For permeability measurement of the caco-2 monolayer, passage of Fluorescein sulfonic acid (FS, 478 Da) from the apical to basolateral compartment in the Transwell chamber was determined. FS is lipophobic and highly-charged at a physiological pH and therefore considered cell impermeable (18). Paracellular passage of FS over the mucosal monolayer from the apical to basolateral compartment therefore indicates impairment of mucosal barrier. FS (200 μg/ml) was administered into the apical compartment of the Transwell chamber together with the drugs diluted in colorless DMEM (with L-glutamine, 3.7g/L NaHCO3 and 10% FCS, 1 mM sodium pyruvate, 1% penicillin/streptomycin).

After 48 h FS concentration was measured in both compartments with a fluorescence spectrophotometer (excitation wavelength of 485 nm and emission wavelength of 528 nm) and the ratio of basaolateral to apical FS concentration was calculated. Data are presented as percentage of the control. This prolonged incubation time allows the detection of FS translocation due to short-term disturbances of barrier integrity as well as potential long-term effects of the used substances.

### Effect of iloprost and nitroglycerin on the permeability of the Caco-2 monolayer

To test the effect of iloprost and nitroglycerin on FS translocation, iloprost (0.1 or 1 μg/ml) or nitroglycerin (25 or 250 μg/ml) were administered together with FS to the apical compartment (*n* = 6 per group). The used concentrations were adopted from the concentrations that have previously been administered in dogs *in vivo* (2). The high concentrations correspond to the concentrations applied to the mucosa. The lower dose was chosen based on a presumed dilution in the stomach. The solvent (NaCl) was used as negative and 2.5 mM ethylene glycol-bis(beta-aminoethyl ether)-N,N,N',N'-tetraacetic acid (EGTA) as positive control. EGTA is a calcium chelator that induces intracellular dissociation of tight junctions (21).

Additionally, two different positive control groups were employed: 1. Triton 2% was used to induce cell death and hence failure of mucosal barrier, 2. 10 mM sodium nitroprusside (SNP). In a similar experimental model, SNP has been shown to induce only a moderate damage to barrier function (22).

### Statistical analysis

GraphPad Prism v6.01 (GraphPad Software, Inc., USA) was used for statistical analysis. Normal distribution was tested with Kolmogorov-Smirnov normality test. Differences were assessed with paired *t*-test (intervention vs. baseline; normally distributed data) and Kruskal-Wallis—Test followed by Dunn's multiple comparison test (not normally distributed data). Data are shown as mean ± SD or median + whiskers (min/max).*P* < 0.05 was considered significant.

## Results

### Effect of iloprost and nitroglycerin on mitochondrial function

**Figure 1:**
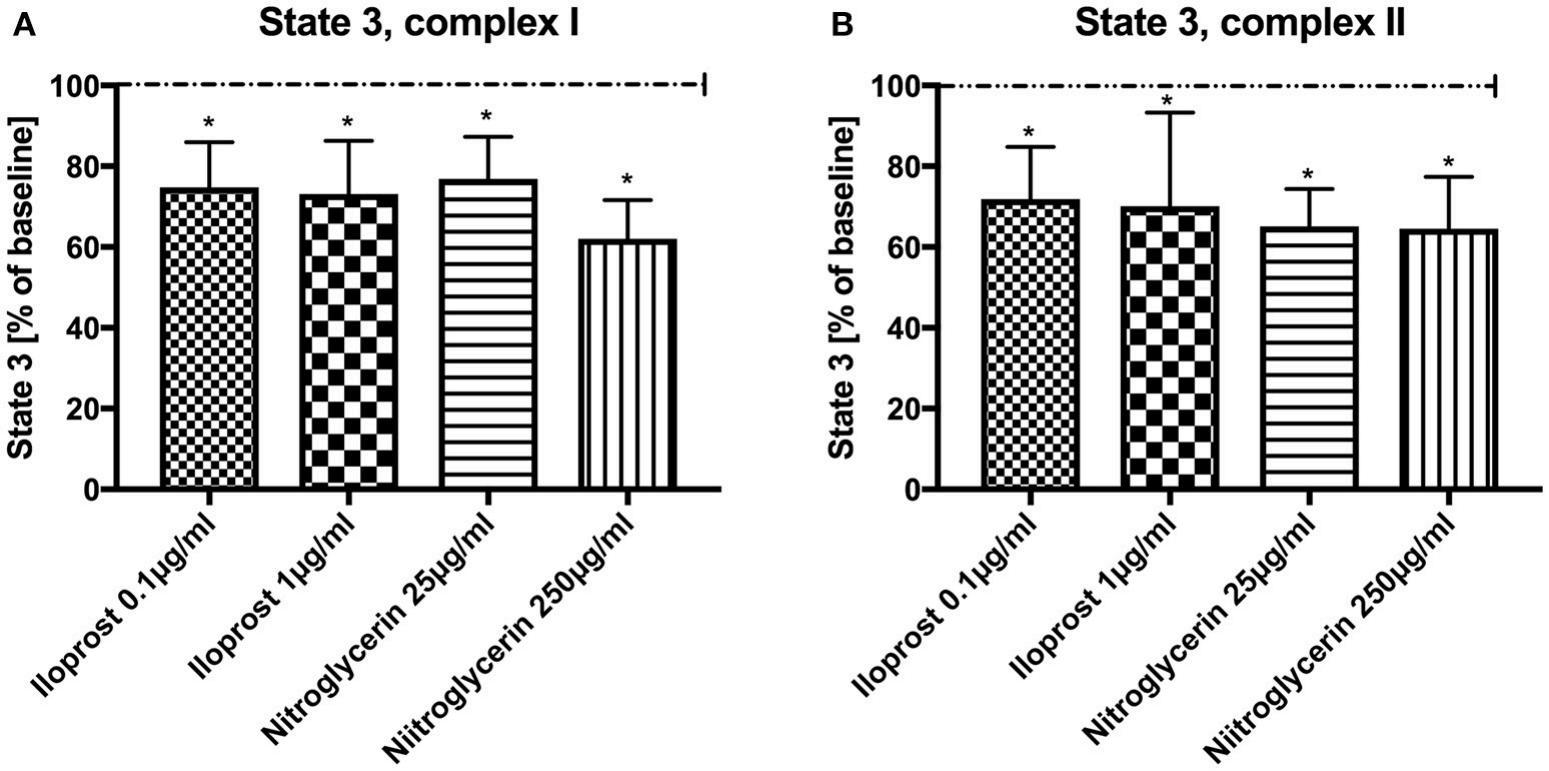
Effect of iloprost (0.1 μg/ml and 1 μg/ml) and nitroglycerin (25 μg/ml and 250 μg/ml) on maximal mitochondrial respiration (State 3) after stimulation through complex I **(A)** and II **(B)** shown as percentage of the baseline value (determined before incubation with iloprost/nitroglycerin). Data are shown as mean ± SD, ^*^p < 0.05 vs. control, *n* = 8.

Absolute values and variability under the employed experimental conditions are listed in Table 1 (baseline conditions). Both concentrations of iloprost and nitroglycerin (iloprost 0.1 and 1 μg/ml, nitroglycerin 25 and 250 μg/ml) reduced the maximal mitochondrial respiration in State 3 by stimulation via both complexes vs. baseline (100%): complex I [iloprost 1 μg/ml: 73 ± 13%^*^, nitroglycerin 250 μg/ml: 62 ± 10%^*^, iloprost 0.1 μg/ml: 75 ± 11%^*^, nitroglycerin 25 μg/ml: 77 ± 11%^*^ (Figure 1A) and complex II (iloprost 1 μg/ml: 70 ± 23%^*^, nitroglycerin 250 μg/ml: 65 ± 13%^*^, iloprost 0.1 μg/ml: 72 ± 13%^*^, nitroglycerin 25 μg/ml: 65 ± 9%^*^ (Figure 1B)].

**Table 1:** Baseline values.

	**Mean**	**S.D**.	***n***
**Baseline values**
State 3 complex I [nmol/min/mg]	2.15	0.44	16
ADP/O complex I	1.47	0.46	16
State 3 complex II [nmol/min/mg]	2.87	0.80	16
ADP/O complex II	1.29	0.37	16

*Absolute values and variability (indicated by S.D.) as prevailing under baseline conditions (pooled baseline data)*.

**Figure 2:**
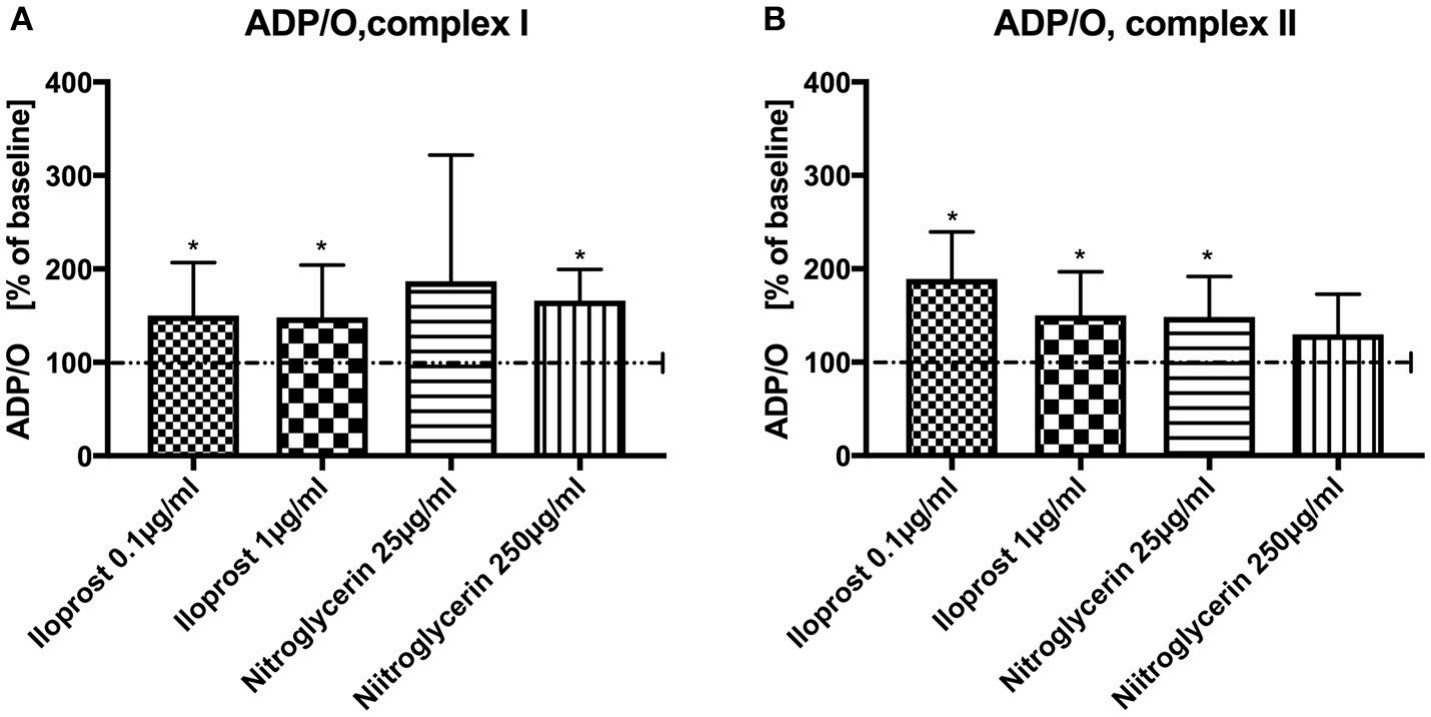
Effect of iloprost (0.1 μg/ml and 1μg/ml) and nitroglycerin (25 μg/ml and 250μg/ml) on ADP/O ratio after stimulation via complex I **(A)** and II **(B)**. Iloprost/Nitroglycerin is shown as percentage of the baseline value. Data are shown as mean ± SD, ^*^p < 0.05 vs. control, *n* = 8.

Iloprost increased ADP/O ratio after stimulation via both complexes and in both concentrations vs. baseline (100%) (iloprost 1 μg/ml, complex I: 148 ± 56%^*^, complex II: 150 ± 47%^*^), iloprost 0.1 μg/ml, complex I: 150 ± 57%^*^, complex II: 189 ± 51%^*^) (Figures 2A,**B**). Nitroglycerin increased ADP/O ratio in the high concentration (250 μg/ml) after stimulation via complex I (100%) (166 ± 34%^*^) and in the lower concentration (25 μg/ml) via complex II vs. baseline (149 ± 43%^*^) (Figure 2A,B).

### Effect of iloprost and nitroglycerin on barrier function

**Figure 3:**
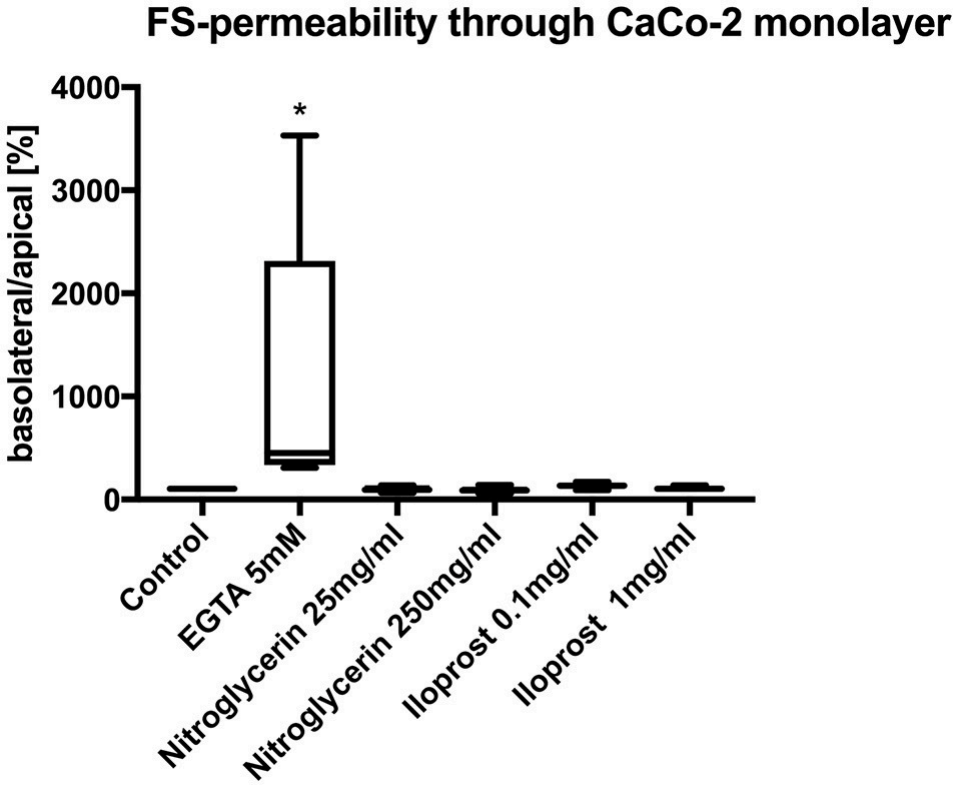
Relative translocation of FS over the epithelial barrier from the apical to the basolateral compartment after incubation with iloprost or nitroglycerin compared to the control group. 5 mM EGTA served as the positive control. Data are shown as percentage of the control value (median/min/max. ^*^p < 0.05 vs. control, *n* = 6).

Neither the application of high-dose nitroglycerin nor low-dose nitroglycerin had any effect on FS translocation (control: 100%, nitroglycerin 25 μg/ml (median/min/max): 95.0/66.7/137.9%, nitroglycerin 250 μg/ml: 87.1/59.3/142.9%) (Figure 3).

Similarly, both concentrations of iloprost had no influence on the FS translocation (control 100%, iloprost 0.1 μg/ml: 135.4/91.0/172.9%, iloprost 1 μg/ml: 104/9/137.9%) (Figure 3). EGTA led to a higher FS-translocation (451.7/309.3/3531%^*^) compared to control. SNP and triton induced massive FS-translocation (360.5/144.4/10955% and 11888/8307/16028%, respectively).

## Discussion

The aim of the present study was to analyze the effect of iloprost and nitroglycerin on colonic mitochondrial function and the direct impact of both drugs on mucosal barrier integrity.

We studied the effect of iloprost and nitroglycerin on mitochondrial function (rat colon homogenates) and on barrier function (Caco-2 monoalyers) using two different drug concentrations: (nitroglycerin 25 μg/ml and 250 μg/ml and iloprost 0.1 μg/ml and 1 μg/ml). The chosen concentrations are based on our previous *in vivo* study with dogs (2). The higher concentrations correspond to those used for local therapy *in vivo* and the 10 times lower concentrations reflect a potential diluting effect in the stomach

The main results of this study are:

1st Iloprost and nitroglycerin reduce the maximal mitochondrial respiration in colon homogenates. Both drugs improve the efficacy of oxidative phosphorylation.

2nd Neither iloprost nor nitroglycerin directly influence mucosal barrier integrity.

Iloprost and nitroglycerin reduced maximal mitochondrial respiration (State 3) to a similar extent at both concentrations. The effect of iloprost on colonic mitochondrial function (tissue homogenate) is different from that on hepatic mitochondrial function (isolated mitochondria, after hypothermic preservation) where iloprost (0.1 μg/ml) had no effect on state 3 (9). In cardiac mitochondria, after ischemia and reperfusion, iloprost (0.01 and 0.1 μg/ml) even enhanced state 3 (23). It confirms the theory, that the changes in mitochondrial function are tissue-specific, stimulus–dependent and differ strongly under different pathophysiological conditions (7, 8).

We are the first to investigate mitochondrial function in colon and to demonstrate reduction of the maximal mitochondrial respiration. Still, it remains unclear whether the reduction in respiration is rather protective or deleterious for the organism. To address this issue, we considered the impact on the efficacy of mitochondrial respiration.

Iloprost improved the efficacy of oxidative phosphorylation (ADP/O-ratio) acting through both complexes and at both concentrations. In hepatic mitochondria, the same concentration of iloprost (0.1 μg/ml) improved ADP/O-ratio only by stimulation through complex II (9). In cardiac mitochondria, the effect of iloprost was shown to be concentration dependent. A lower concentration of iloprost (0.001 μg/ml) did not change the efficacy of oxidative phosphorylation, while it was improved at higher concentrations (0.01 and 0.1 μg/ml). In this study, the respiratory chain was stimulated only through complex I (23). Thus, the effects of iloprost on colonic mitochondria are similar to those observed in the heart.

In the present study, nitroglycerin improved the efficacy of oxidative phosphorylation but the effect differed between both concentrations: the lower concentration increased ADP/O after stimulation through complex II and the higher through complex I. This observation is partially in line with observations in other organs (liver) where nitroglycerin acts through complex I but NO through both complexes (24). Why the effects of nitroglycerin on colonic mitochondrial function differs between both concentrations remains unclear.

In summary, both iloprost and nitroglycerin improve the efficacy of oxidative phosphorylation in colon homogenate. As a consequence, the amount of produced ATP remains unchanged when oxygen consumption is reduced. Thus, the reduction in respiration can be considered rather protective than deleterious for the organism.

Another aim of this study was to examine the direct effects of iloprost and nitroglycerin on mucosal barrier integrity. To address this issue, we employed a well established *in vitro* model of the intestinal mucosal barrier which allows us to analyze direct effects independent of microcirculation and changes in oxygen supply. To validate this experimental setup, we employed positive and negative controls. EGTA was used to show that our model is sensitive enough to detect minor damage to tight junctions. Additionally, SNP and triton were employed to simulate moderate damage and complete loss of barrier function, respectively, which was confirmed by the extensive FS translocation (22). One may critically discuss the chosen 48 hour incubation period. We expected a short-term disturbance of intestinal barrier with translocation of FS. However, long-term effects cannot be excluded and this observation period allows us to detect potential short- and long-term effects on barrier integrity.

Nitroglycerin and iloprost had no effect on barrier function. FS translocation after incubation with both drugs led to FS translocation similar to the controls. This indicates that neither nitroglycerin nor iloprost exert any direct, harmful effects on mucosal barrier integrity.

This study has certain limitations. To address both questions about mitochondrial function and barrier function we used *in vitro* models. Concerning barrier function this does not take into account possible effects that might occur in a living organism like leucocyte adhesion and inflammatory reactions. The method for measuring the mitochondrial function in colon homogenates was newly established. To achieve this, a short incubation of the tissue with trypsin is necessary before homogenization. The appropriate quality of the preparation was verified by the integrity of the inner and outer mitochondrial membrane.

In this study we didn't examine non-ETS respiration [oxygen utilization by other enzymes independent of the electron transport system (ETS)]. However, non-ETS respiration would affect all measurements equally and therefore differences due to the used drugs can still reliably be detected. Nevertheless, it could be regarded as a potential limitation of the study.

This study also has certain strengths. We used well-established models to estimate mitochondrial respiration and barrier function (19, 25). The aim of this study was to analyze direct effects of iloprost and nitroglycerin on colonic mitochondrial function and on barrier integrity independent of systemic changes like oxygen supply. This can only be analyzed in isolated *in vitro* models. The *in vitro* measurement of mitochondrial function in tissue homogenates has an advantage over the measurement in isolated mitochondria because the isolation process might lead to the loss of a specific fraction of mitochondria, and cause additional damage (e.g., uncoupling) (25).

The results of this *in vitro* study cannot be directly transferred into humans. However, there is a lot of evidence that mitochondrial function differs between different organs, with little evidence that mitochondrial function in the same organ differs between different species (7). Our animal data suggests that iloprost and nitroglycerin might be safely applied in the used concentrations because they didn't cause any systemic side effects and showed positive effect on the local gastric oxygenation and nitroglycerin improved the barrier function (1). Improved intestinal oxygenation could be possibly explained through reduced maximal mitochondrial respiration and improved efficacy of oxidative phosphorylation Our *in vitro* data supports the conclusion and extend our knowledge about a possible mode of action of these both drugs. Local application of the drugs, having no systemic side effect and no direct negative influence on the barrier function but influencing positively the mitochondrial function could therefore offer a novel, promising therapeutic option in the human medicine.

## Ethics statement

The study was carried out in accordance with the Guide for the Care and Use of Laboratory Animals of the National Institutes of Health. The protocol was approved from the Animal Ethics Committee of the University of Duesseldorf, Germany,

## Author's note

Preliminary data of this manuscript were presented on the following conferences and partly appeared as conference papers.

Herminghaus A, Vollmer C, Eberhardt R, Truse R, Schulz J, Bauer I, Picker O. Iloprost und Nitroglycerin vermindern den mitochondrialen O_2_-Verbrauch und steigern die Effektivität der oxidativen Phosphorylierung im Colonhomogenat ohne Einfluss auf die Barriereintegrität von Caco-2-Monolayern *in vitro*. Wissenschaftliche Arbeitstage der DGAI, Würzburg, 2017 Anästh Intensivmed 2017;58:384.

Herminghaus A, Vollmer C, Eberhardt R, Truse R, Schulz J, Bauer I, Picker O. Iloprost und Nitroglycerin vermindern den mitochondrialen O_2_-Verbrauch und steigern die Effektivität der oxidativen Phosphorylierung im Colonhomogenat ohne Einfluss auf die Barriereintegrität von Caco-2-Monolayern *in vitro*. Deutscher Anästhesikongress, Nürnberg, 2017 Anästh Intensivmed 2017;58:S 367.

Herminghaus A, Vollmer C, Eberhardt R, Truse R, Schulz J, Bauer I, Picker O. Nitroglycerin and iloprost improve mitochondrial function in colon homogenate without altering the barrier integrity of Caco-2 monolayers. European Shock Society Congress, Paris, 2017.

## Author contributions

AH acquisition of data, conception and design, analysis and interpretation of data, and drafting the article. RE acquisition of data, analysis and interpretation of data, and revising the article. RT and JS analysis and interpretation of data, and revising the article. IB and OP conception and design, analysis and interpretation of data, and revising the article. CV acquisition of data, conception and design, analysis and interpretation of data, and revising the article. All authors read and approved the final manusript.

### Conflict of interest statement

The authors declare that the research was conducted in the absence of any commercial or financial relationships that could be construed as a potential conflict of interest.
